# Effects of a calorie-restricted dietary intervention on weight loss and gut microbiota diversity in obese patients with sleep deprivation

**DOI:** 10.1007/s40519-023-01609-5

**Published:** 2023-10-04

**Authors:** Surong Wen, Yaojun Ni, Yuhong Dai, Ziyu Liu, Xiaoqing Wang, Jie Zhang, Weinan Yu, Wen Hu

**Affiliations:** 1 Department of Endocrinology, Huai’an Hospital Affiliated to Xuzhou Medical University and Huai’an Second People’s Hospital, Huai’an, 223002 Jiangsu China; 2 Department of Cardiothoracic Surgery, Hospital Affiliated to Nanjing Medical University and Huai’an First People’s Hospital, Huai’an, 223001 Jiangsu China

**Keywords:** Sleep deprivation, Gut microbiota, Calorie restricted diet, Obesity

## Abstract

**Objectives:**

This study aimed to investigate the effects of a calorie-restricted dietary (CRD) intervention on weight and gut microbiota diversity in obese patients with sleep deprivation (SD).

**Methods:**

Twenty obese patients were divided into a sleep deprivation group (SD group, *n* = 10) and a nonsleep deprivation group (NSD group, *n* = 10), both of which underwent a CRD intervention for 12 weeks. Measurement of anthropometric parameters, biochemical examinations and gut microbiota detection were performed at baseline and at the end of week 12. Mi Smart Bands 1 (Standard Option) were used to monitor sleep and exercise.

**Results:**

(1) The CRD intervention improved body weight (BW), waist circumference (WC), blood pressure (BP), basal metabolic rate (BMR), body fat content (BFC), and insulin resistance index (HOMA-IR) in all obese patients. (2) In the NSD group, BW, BFC, VFA (visceral fat area), BMR and total cholesterol (TC) were significantly reduced after the CRD intervention (*P* < 0.05). (3) The alpha diversity of the gut microbiota remained unchanged after the intervention in the two groups. (4) There was a negative correlation between Mollicutes and BMR in the NSD group.

**Conclusions:**

The effects of a CRD intervention weaken on weight loss and the metabolism of blood lipids may be weakened by SD. The abundance of Mollicutes bacteria may be related to weight loss after a CRD intervention in obese patients.

**Level of evidence:**

III, prospective cohort study.

**Supplementary Information:**

The online version contains supplementary material available at 10.1007/s40519-023-01609-5.

## Introduction

Obesity is a common disease that threatens the physical and mental health approximately of about 39% of people around the world [1]. It is also a risk factor for many diseases, such as cancers, cardiovascular diseases, endocrine system diseases and digestive system diseases. Safe and effective weight loss can reduce the risk of obesity-related diseases and even decrease all-cause mortality in obese patients [2]. Currently, many strategies have been developed to treat obesity, such as dietary intervention, exercise, drugs and surgery, and dietary intervention is the first step in the process of obesity management [3]. The calorie-restricted diet (CRD) recommended by the Chinese Nutrition Society is a nutritionally balanced, safe and feasible strategy with a favourable weight-loss losing effect [4]. However, the weight-loss losing effect varies among individuals, and the specific mechanism for this variation is still unknown.

Sleep deprivation (SD) was defined as a forced reduction in sleep duration (generally < 6 h within 24 h) for various reasons [5]. Sleep deprivation can cause a series of physiological and psychological changes, leading to a variety of adverse consequences, such as cognitive decline, metabolic disorders, and obesity [6]. In addition, SD may also affect the diversity of gut microbiota, which may compromise weight loss [7, 8]. Therefore, this study was undertaken to investigate the effects of CRD intervention on weight loss and gut microbiota diversity in obese patients with SD.

## Materials and methods

### Subjects

Twenty subjects with simple obesity were recruited from Huai'an Second People’s Hospital between September 2018 and December 2019. The study protocol for obese subjects was the Ethics Committee of Huai’an Hospital, Xuzhou Medical University and Huai’an Second People’s Hospital (Huai’an city, Jiangsu Province, China). All subjects signed informed consent forms before the study. This study was registered in the Clinical Trial Registration Center (No: ChiCTR1800015171).

The inclusion criteria were as follows: (1) older than 18 years but younger than 50 years; (2) body mass index (BMI) ≥ 28.0 kg/m^2^; and (3) psychological assessments and CRD intervention. Exclusion criteria were as follows: (1) subjects were diagnosed with secondary obesity; (2) subjects had gastrointestinal diseases or malabsorption; (3) subjects were previously diagnosed with severe liver, kidney, heart or lung dysfunction, hypothyroidism or malignant tumour; (4) subjects were treated with glycaemic drugs, lipid-lowering drugs, weight loss drugs, glucocorticoids and other drugs affecting metabolism within the last 4 weeks; (5) subjects received weight-loss surgery before study; (6) subjects were previously diagnosed with mental disorders, and the inability to provide self-reported data and informed consent; (7) subjects were pregnant or breast-feeding; (8) subjects had a history of alcohol abuse (amount of alcohol consumed per week was ≥ 140 g for men and ≥ 70 g for women); (9) subjects were previously diagnosed with physical dysfunction; (10) subjects participated in other weight loss programs or their body weight decreased by more than 5% in the past 6 months; and (11) subjects had poorly controlled severe hypertension (systolic blood pressure [SBP] ≥ 140 mmHg and diastolic blood pressure [DBP] ≥ 90 mmHg).

All the subjects were divided into two groups: the SD group (*n* = 10) and the NSD group (*n* = 10). In the SD group the daily sleep time was less than 6 h, while in the NSD group, the time was longer than 7 h and shorter than 9 h [9]. After a 12-week follow-up, 2 subjects (one in the SD group, another in the NSD group) failed to complete the intervention and another 2 subjects (one in the SD group, another in the NSD group).were treated with lipid-regulating and uricase-lowering drugs or experienced a traffic accident. Thus, they were excluded from this study. Finally, sixteen subjects (NSD group, *n* = 8; SD group, *n* = 8) completed this study. There were no significant differences in sex, age, body weight (BW), body mass index (BMI) or biochemical parameters between the two groups at baseline.

## General information collection

After the questionnaire survey and psychological assessment, all subjects underwent a intervention CRD combined with appropriate exercise (at least 10,000 steps per day) for weight loss for 12 weeks.

In the CRD intervention, the daily energy intake was 20–25 kcal/kg per unit standard weight [9]. The standard weight (kg) = height (cm) − 105. The proportions of energy at breakfast, lunch and dinner were 30%, 40% and 30%, respectively. The proportions of carbohydrate, protein and fat in the diet were 40%, 20% and 40%, respectively. Meal replacement bars (Guangzhou NanDaFeiTe Nutrition Health Consulting Co. Ltd) were taken twice daily (before breakfast and before dinner). During the study, the Mi Band was used to monitor sleep and activities (the validity of the Mi Band met the requirements for the study). Body shape assessment, biochemical examination, liver colour ultrasonography and gut microbiota detection were performed at baseline and 12 weeks after the CRD intervention.

### Questionnaire

Baseline demographic characteristics, lifestyle information and medical history were collected by the trained investigators through standard questionnaires. Current smokers were defined as subjects who smoked at least one cigarette per day over the past month. Drinking was defined as drinking at least once weekly for nearly 1 month. Mental health status was assessed with the Mental Health Assessment Scale (Symptom Checklist 90, SCL-90) [10].

### Data collection

Body shape parameters, including basal metabolic rate (BMR), body fat content (BFC), body fat percentage (BFP), visceral fat area (VFA) and BW, were measured with the Korean JAWON body composition analyser ioi353. BMI was calculated as weight (kg) divided by height squared (m^2^). Waist circumference (WC) was measured with a nonstretchable tape over the unclothed abdomen at the narrowest point between the lowest rib and the iliac crest. Two measures were obtained, and the mean (centimetres) was calculated for analyses. Blood pressure (BP) was consecutively measured three times (OMRON Model HEM-752 FUZZY, Omron Company, Dalian, Liaoning, China), and the mean was calculated for further use.

### Blood detection and hepatic ultrasonography

After overnight fasting (at least 8 h), blood samples were collected from the antecubital vein at 8:00 a.m ~ 10:00 a.m. Fasting plasma glucose (FPG), total cholesterol (TC), triglycerides (TG), creatinine (Cr), urea nitrogen (BUN), uric acid (UA), fasting insulin (FIns), glutamate–pyruvate transaminase (AST) and alkaline phosphatase (ALP) were measured with standardized protocols. HbA1c was measured by high-performance liquid chromatography (Variant II and D-10 Systems, Bio-Rad Laboratories Inc, Hercules, CA, USA).

Insulin resistance index (HOMA-IR) = FPG (mmol/l) × FIns (μU/ml)/22.5 [11]. The estimated glomerular filtration rate (eGFR) was calculated using the Chronic Kidney Disease Epidemiology Collaboration (CKD–EPI) equation [12].

Hepatic ultrasonography was performed in all subjects by an experienced clinician using an Aloka Prosound α 6 probe (Hitachi Aloka, Japan) with a 3-MHz probe under the same working conditions and operating standards. Mild, moderate and severe fatty liver disease was determined according to the Doppler spectrum of hepatic vein pulse and liver haemodynamics [13].

### Detection of the gut microbiota

Faeces (as large as the size of soybean) (approximately 2 g) were collected in the morning from subjects after fasting and stored separately in a sterile container at − 80 °C at baseline and after the 12-week CRD intervention. Complete genomic DNA was extracted and purified using the QIAamp DNA Stool Mini Kit (QIAGEN, Hilden, Germany) according to the manufacturer’s instructions. Statistical analysis was conducted based on taxonomic information.

### Statistical analysis

The quantitative data are expressed as the means ± standard deviations; qualitative data are expressed as numbers or percentages. Paired *t* tests were used to compare the data before and after the intervention in the same group. Comparisons between groups were performed using Student’s *t* test for quantitative data and the chi-square test for qualitative data. Stamp software was used to analyse the differences in gut microbiota distribution. The Kruskal–Wallis test was used to compare the differences in intestinal bacteria between groups, and Spearman correlation analysis was used to analyse the correlation between clinical indicators and host flora. SD_0_ and NSD_0_ represented the data at baseline in the SD group and NSD group, respectively. SD_12_ and NSD_12_ represent the data at the end of the 12-week CRD intervention in the SD group and NSD group, respectively. A value of *P* < 0.05 was considered to indicate statistical significance.

## Results

### Characteristics of subjects before and after the 12-week CRD intervention

The WC, SBP, DBP, BFC, BW, BMR, HOMA-IR, TG and ALT in all subjects significantly decreased after the CRD intervention compared to those at baseline (Table 1).

**Table 1:** Characteristics of subjects before and after the 12-week CRD intervention

Variates	SD (*n* = 8)				NSD (*n* = 8)						Total (*n* = 16)			
	Before CRD intervention	After CRD intervention	*t*_*a*_	*P*_*a*_	Before CRD intervention	After CRD intervention	*t*_*b*_	*t*_*0*_	*P*_*b*_	*P*_*0*_	Before CRD intervention	After CRD intervention	*t*_*c*_	*P*_*c*_
Sex (Male, %)	7 (87.5%)	–	–	–	5 (62.5%)	–	–	–	–	–	12 (75%)	–	–	–
Age (years)	38.13 ± 2.44	–	–	–	34.00 ± 3.78	–	–	− 1.484	–	0.160	34.00 ± 1.34	–	–	–
BMI (kg/m^2^)	29.85 ± 1.71	29.33 ± 1.82	1.626	0.148	31.29 ± 2.49	29.30 ± 6.25	1.264	− 1.347	0.247	0.199	30.57 ± 2.19	30.52 ± 4.45	1.575	0.136
WC (cm)	100.73 ± 6.14	98.60 ± 6.76	3.694	0.008	101.61 ± 8.52	96.14 ± 11.26	3.103	− 0.239	0.017	0.815	101.17 ± 7.19	97.37 ± 9.06	3.818	0.002
BW (kg)	85.88 ± 8.76	83.85 ± 9.30	2.898	0.023	88.00 ± 13.16	79.23 ± 14.52	3.895	− 0.380	0.006	0.709	86.94 ± 10.85	81.54 ± 12.02	3.766	0.002
SBP (mmHg)	128.38 ± 7.27	120.63 ± 9.44	3.025	0.019	122.75 ± 11.85	118.00 ± 10.50	3.366	1.144	0.012	0. 272	125.56 ± 9.93	119.31 ± 9.74	4.267	0.001
DBP (mmHg)	91.38 ± 9.41	82.88 ± 5.84	4.007	0.005	87.25 ± 8.24	81.50 ± 5.32	3.369	0.933	0.008	0. 367	89.31 ± 8.81	82.19 ± 5.44	5.400	< 0.001
BFC (kg)	27.11 ± 3.28	26.56 ± 2.78	0.805	0.447	29.36 ± 5.44	24.11 ± 6.41	2.909	− 1.001	0.023	0.334	28.24 ± 4.50	25.34 ± 4.94	2.607	0.020
BFP (%)	31.64 ± 2.95	31.33 ± 2.25	0.368	0.724	33.46 ± 4.74	30.36 ± 5.38	2.001	− 0.925	0.085	0.371	32.55 ± 3.93	30.84 ± 4.02	1.842	0.085
BMR (kcal)	1501.75 ± 149.36	1476.25 ± 146.63	3.599	0.009	1477.38 ± 229.98	1427.00 ± 228.02	5.994	0.251	0.001	0.805	1489.56 ± 187.75	1451.63 ± 187.75	6.113	< 0.001
VFA (cm^2^)	135.00 ± 19.31	138.25 ± 23.34	-0.638	0.544	145.00 ± 39.92	108.88 ± 35.29	2.088	− 0.638	0.075	0.534	140.00 ± 30.73	123.56 ± 32.64	1.630	0.124
HbA1c (%)	5.39 ± 0.36	5.24 ± 0.58	0.644	0.540	5.53 ± 0.41	5.29 ± 0.31	1.610	− 0.709	0.168	0.490	5.46 ± .38	5.26 ± .45	1.449	0.168
HOMA-IR	2.68 ± 1.19	2.15 ± 0.84	1.100	0.308	3.44 ± 1.83	1.91 ± 1.78	2.648	− 0.979	0.033	0.344	1.54 ± 0.39	1.35 ± 0.34	2.671	0.017
TC (mmol/l)	4.56 ± 0.66	4.62 ± 0.77	− 0.482	0.645	4.91 ± 1.18	4.16 ± 0.95	2.867	− 0.764	0.024	0.457	4.86 ± 0.96	4.38 ± 0.87	1.967	0.068
TG (mmol/l)	1.71 ± 1.33	1.20 ± 1.01	2.520	0.040	1.50 ± 1.11	0.94 ± 0.45	1.798	0.336	0.115	0.742	1.62 ± 1.19	1.07 ± 0.77	2.980	0.009
ALT (U/l)	26.75 ± 8.46	19.75 ± 7.32	2.306	0.055	44.50 ± 35.43	22.50 ± 11.02	1.954	− 1.378	0.092	0.206	35.63 ± 26.52	21.13 ± 9.15	2.434	0.028
AST (U/l)	23.38 ± 6.80	19.38 ± 2.67	1.508	0.175	27.00 ± 15.10	18.75 ± 6.61	1.582	− 0.619	0.158	0.546	25.19 ± 11.47	19.06 ± 4.88	2.127	0.050
ALP (U/l)	60.75 ± 20.53	72.88 ± 27.2	− 0.860	0.418	76.75 ± 29.68	92.87 ± 21.94	− 1.278	− 1.254	0.242	0.230	68.75 ± 26.01	82.88 ± 26.03	1.543	0.144
BUN (mmol/l)	5.28 ± 0.94	4.77 ± 1.05	0.788	0.457	5.85 ± 1.09	4.93 ± 1.52	1.852	− 1.132	0.107	0.277	5.57 ± 1.03	4.85 ± 1.27	1.803	0.092
Cr (μmol/l)	73.12 ± 18.67	72.11 ± 11.95	0.189	0.855	67.94 ± 20.86	63.08 ± 14.37	1.096	0.524	0.309	0.609	70.53 ± 19.31	67.59 ± 13.60	0.865	0.401
eGFR (ml/min)	110.97 ± 16.36	113.42 ± 10.61	− 0.468	0.654	114.32 ± 18.62	120.65 ± 8.79	− 0.854	0.383	0.243	0.708	112.64 ± 17.02	117.03 ± 10.1	1.386	0.186

All variables are expressed as n (%) for qualitative data or mean ± standard deviation for quantitative data. *t*_*a*_*,* the *t* value of the change in each parameter before and after intervention in the NSD group. *t*_*0*_, the *t* value of baseline parameters between the SD group and NSD group. *t*_*c*_, the *t* value of the change in each parameter before and after intervention in all subjects. *P*_*a*_, the difference in the change in each parameter before and after intervention in the SD group. *P*_*b*_, the difference in the change in each parameter before and after the intervention in the NSD group. *P*_*0*_, the difference in baseline parameters between the SD group and NSD group. *P*_*c*_, the difference in the change in each parameter before and after intervention in all subjects. NSD, nonsleep deprivation group. SD, sleep deprivation group. BMI, body mass index; WC, waist circumference; BW, body weight; SBP, systolic blood pressure; DBP, diastolic blood pressure; BFC, body fat content; BFP, body fat percentage; BMR, basal metabolic rate; VFA, visceral fat area; HbA1c, haemoglobin A1c; HOMA-IR, homeostasis model of assessment for insulin resistance index; TC, total cholesterol; TG, triglycerides; ALT, alanine aminotransferase; AST, aspartate aminotransferase; ALP, alkaline phosphatase; BUN, blood urea nitrogen; Cr, creatinine; eGFR, estimated glomerular filtration rate

At baseline, there were no marked differences in age, sex, BMI, WC, BW, SBP, DBP, BFC, BFP, BMR, VFA, HbA1c, HOMA-IR, HbA1c, TC, TG, ALT, AST, ALP, BUN or eGFR between the SD group and NSD group (Table 1). After CRD intervention, the BW, WC, BMR, SBP and DBP decreased significantly in both groups. However, there were slight declines in TG in the SD group and TC and HOMA-IR in the NSD group (Table 1).

### Changes in the parameters in the two groups before and after the CRD intervention

After the CRD intervention, the mean BFC, VFA, BW, BMR and TC in the NSD group decreased by 4.70 kg, 39.38 cm^2^, 6.74 kg, 24.88 kcal and 0.83 mmol/l, respectively, and these changes were more obvious than those in the SD group. However, there were no significant differences in the changes inBMI, BFP, WC, ALT, AST, ALP, BUN, eGFR, TGs, HOMA-IR, HbA1c, SBP and DBP between the NSD group and SD group after invention (Table 2).

**Table 2:** Changes in the body parameters and biochemical parameters after the CRD intervention

Variates	SD	NSD	*t* value	*P*
n	8	8	–	–
∆BMI (kg/m^2^)	0.53 ± 0.91	1.99 ± 4.45	− 0.911	0.378
∆WC (cm)	2.13 ± 1.63	5.48 ± 4.99	− 1.805	0.107
∆BW (kg)	2.02 ± 1.97	8.76 ± 6.36	− 2.860	0.013
∆SBP (mmHg)	7.75 ± 7.25	4.75 ± 3.99	1.026	0.322
∆DBP (mmHg)	8.50 ± 6.00	5.75 ± 4.40	1.045	0.314
∆BFC (kg)	0.55 ± 1.93	5.25 ± 5.10	− 2.436	0.029
∆BFP (%)	0.31 ± 2.40	3.10 ± 4.38	− 1.578	0.137
∆BMR (kcal)	25.50 ± 20.04	50.38 ± 23.77	− 2.263	0.040
∆VFA (cm^2^)	− 3.25 ± 14.41	36.13 ± 48.94	− 2.183	0.047
∆HbA1c	0.15 ± 0.66	0.23 ± 0.42	− 0.317	0.756
∆HOMA-IR	0.53 ± 1.36	1.53 ± 1.64	− 1.111	0.904
∆TC (mmol/l)	-0.06 ± 0.37	0.77 ± 0.76	− 2.791	0.014
∆TG (mmol/l)	0.051 ± 0.57	0.56 ± 0.881	− 0.135	0.895
∆ALT (U/l)	7.00 ± 8.59	22.00 ± 31.85	− 1.286	0.219
∆AST (U/l)	4.00 ± 7.50	8.25 ± 14.75	− 0.726	0.480
∆ALP (U/l)	− 12.13 ± 39.87	− 16.13 ± 35.69	0.211	0.836
∆BUN (mmol/l)	0.51 ± 1.82	0.92 ± 1.41	− 0.514	0.615
∆Cr (μmol/l)	1.01 ± 15.17	4.86 ± 12.55	− 0.553	0.589
∆eGFR (ml/min)	2.45 ± 14.78	6.33 ± 14.03	− 0.538	0.599

All variables are expressed as n (%) for qualitative data or mean ± standard deviation for quantitative data. NSD, nonsleep deprivation group. SD, sleep deprivation group. BMI, body mass index; WC, waist circumference; BW, body weight; SBP, systolic blood pressure; DBP, diastolic blood pressure; BFC, body fat content; BFP, body fat percentage; BMR, basal metabolic rate; VFA, visceral fat area; HbA1c, haemoglobin A1c; HOMA-IR, homeostasis model of assessment for insulin resistance index; TC, total cholesterol; TG, triglycerides; ALT, alanine aminotransferase; AST, aspartate aminotransferase; ALP, alkaline phosphatase; BUN, blood urea nitrogen; Cr, creatinine; eGFR, estimated glomerular filtration rate. “∆”: difference between baseline level and 12-week level

### Findings from Doppler ultrasonography

At baseline, fatty liver disease was noted in all the subjects in both the SD group and the NSD group. After the 12-week intervention, fatty liver disease was not observed in 87.5% and 37.5% of subjects in the NSD group and SD group, respectively.

### Gut microbiota distribution

**Fig. 1:**
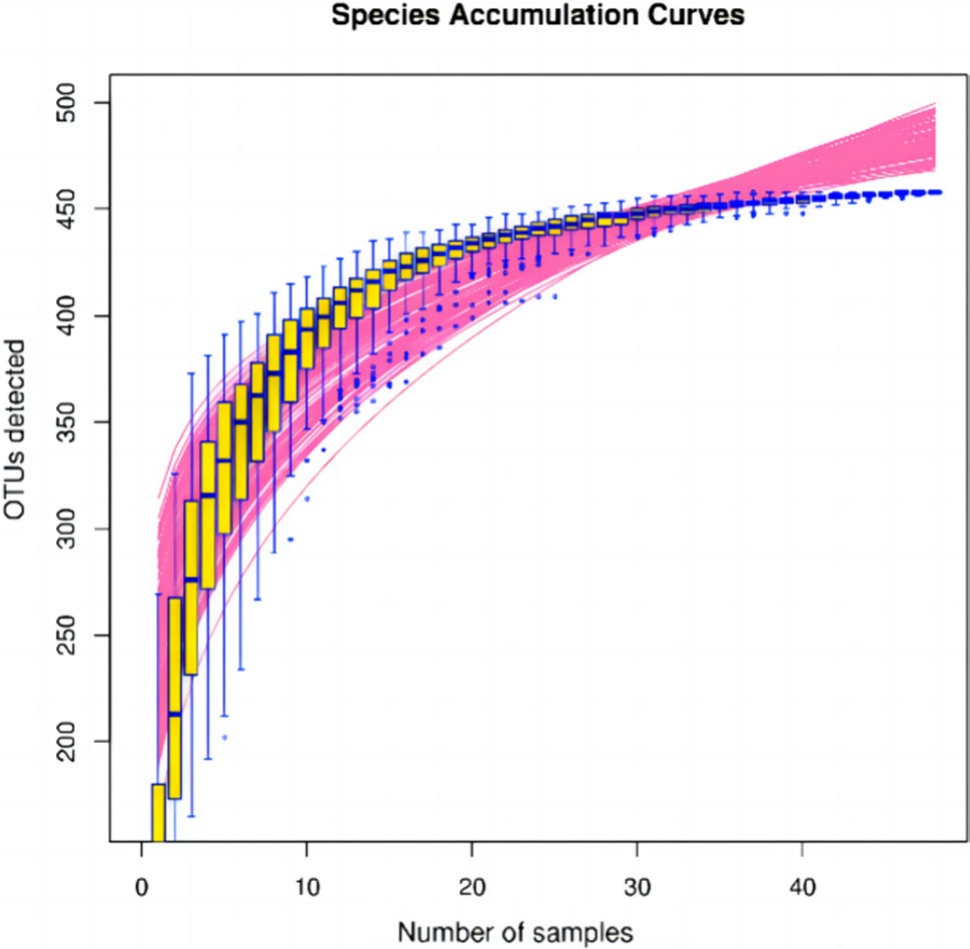
Species accumulation curve of the sequencing depth test. OTU: operational taxonomic units

*Sequencing depth of gut microbiota* the species accumulation curve of the samples was analysed by a random sampling method to detect the sequencing depth. The results showed that the dilution curve tended to flatten when there were 30 sequencing samples, indicating that the depth of this sequencing met the requirements, and the additional amount of sequencing data was not necessary for studying the discovery of OTUs (Fig. 1).

### Gut microbiota in the two groups before and after CRD intervention

**Fig. 2:**
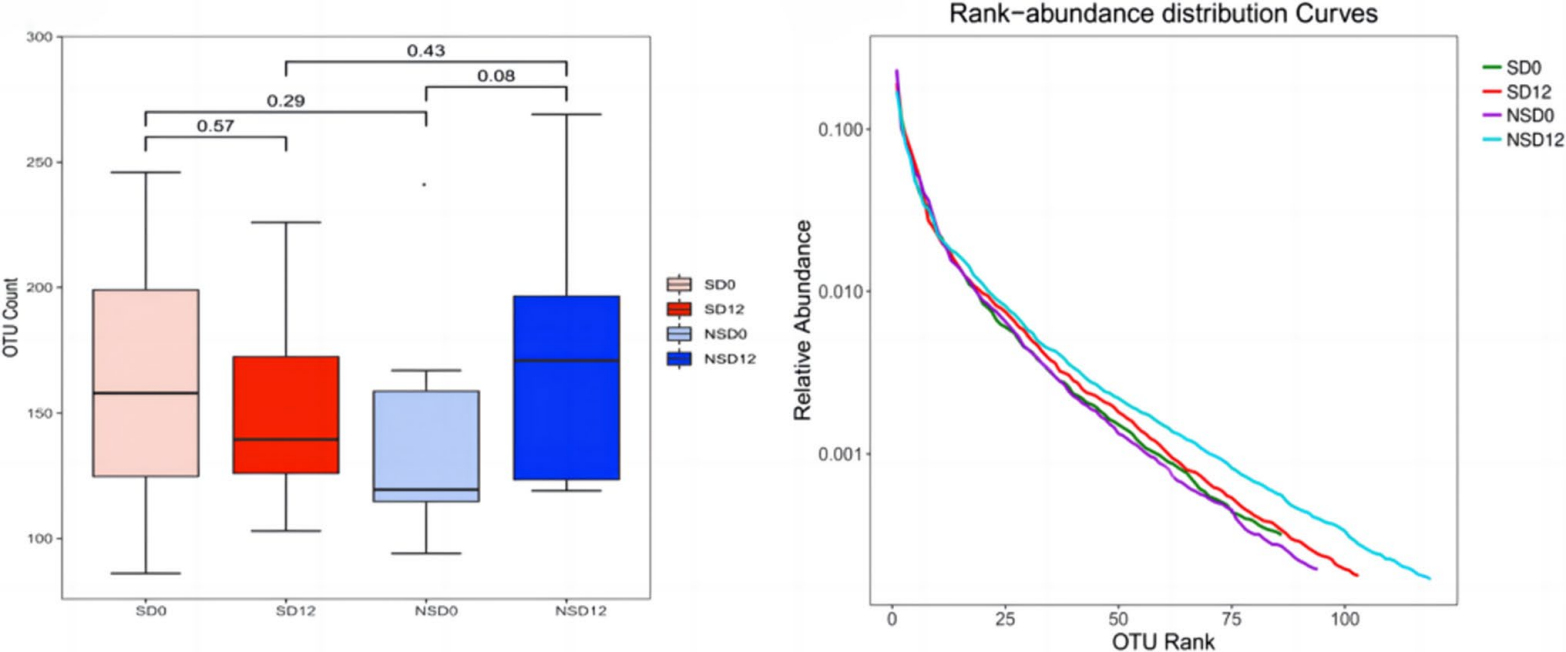
Gut microbiota abundance of the subjects. **A** Gut microbiota abundance alpha diversity between the SD group and NSD group before and after intervention. **B** Curve analysis of gut microbiota and species rank abundance. SD_0_: baseline in the SD group, SD_12_: the end of 12 weeks after CRD intervention in the SD group, NSD_0_: baseline in NSD group, NSD_12_: the end of 12 weeks after CRD intervention in the NSD group

As shown in Fig. 2A, the numbers of OTUs were similar between SD_0_ and SD_12_, NSD_0_ and NSD_12_, SD_0_ and NSD_0_, and SD_12_ and NSD_12_ (*P*SD0–SD12 = 0.57,* P*NSD0–NSD12 = 0.08, *P*SD0–NSD0 = 0.29 and *P*SD12–NSD12 = 0.43). However, as shown in Fig. 2B, after the intervention, the abundance in the NSD group increased gradually, while it decreased in the SD group. Compared with the NSD group, the abundance in the SD group was lower before after CRD intervention, without significance.

### Heatmap analysis of the correlation between gut microbiota abundance and clinical factors before and after intervention in the two groups

**Fig. 3:**
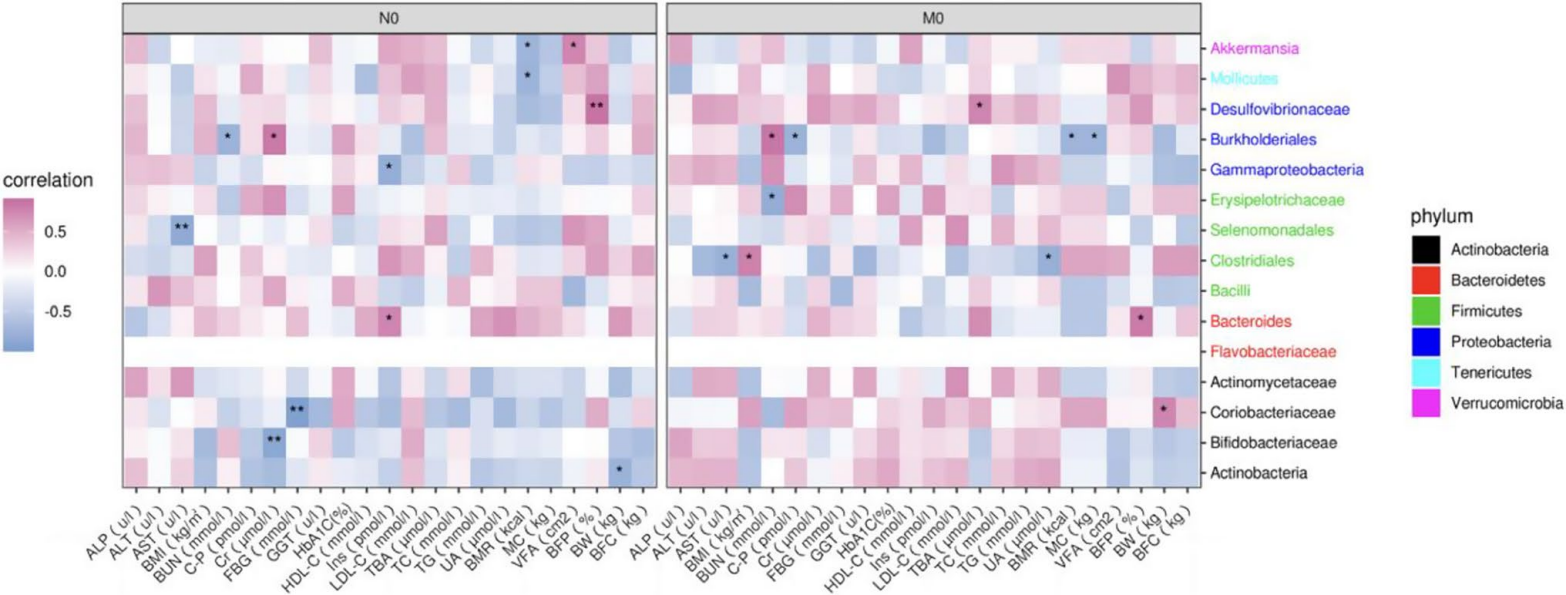
Heatmap analysis of the correlation between the gut microbiota at the phylum level and clinical indicators at baseline. MC, muscle content; C-P, c-peptide; Cr, creatinine; FBG, fasting plasma glucose; GGT, γ-glutamyl Transpeptidase; HDL-C, high-density lipoprotein cholesterol; LDL-C, low-density lipoprotein cholesterol; TBA, total bile acid; UA, uric acid; BMI, body mass index; BFC, body fat content; BFP, body fat percentage; VFA visceral fat area; BW, body weight; WC, waist circumference; BMR, basal metabolic rate; ALT, alanine aminotransferase; AST, aspartate aminotransferase; ALP, alkaline phosphatase; BUN, blood urea nitrogen; TC, total cholesterol; TG, triglycerides; HbA1c, haemoglobin A1c; SD_0_ baseline in the sleep deprivation group, NSD_0_ baseline in nonsleep deprivation group, *P < 0.05, **P < 0.01

At the phylum level, heatmaps were plotted based on the correlation coefficient between the abundance of gut microbiota and clinical indicators. As shown in Fig. 3, at baseline, there was a strong correlation between some gut microbiota and clinical factors in the NSD group. Selenomonadales was negatively related to AST, Bifidobacteriaceae and Coriobacteriaceae was negatively related to FBG. Desulfovibrionaceae was positively related to BFR. There was a negative correlation between Mollicutes and BMR.

However, there was no highly significant association (*P* value > 0.01) in the SD group between the abundance of gut microbiota and clinical factors.

**Fig. 4:**
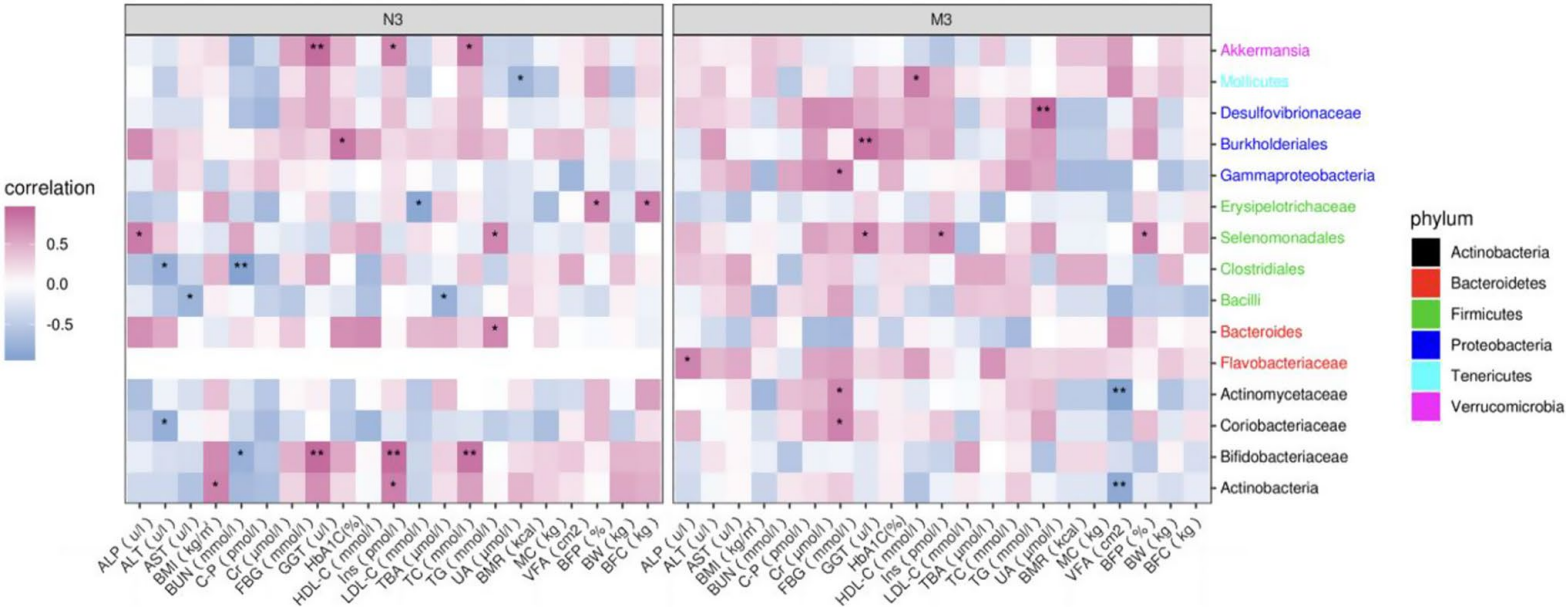
Heatmap analysis of the correlation between gut microbiota at the level of phylum intervention and clinical indicators. MC, muscle content; C-P, c-peptide; Cr, creatinine; FBG, fasting plasma glucose; GGT, γ-glutamyl Transpeptidase; HDL-C, high-density lipoprotein cholesterol; LDL-C, low-density lipoprotein cholesterol; TBA, total bile acid; UA, uric acid; BMI, body mass index; BFC, body fat content; BFP, body fat percentage; VFA visceral fat area; BW, body weight; WC, waist circumference; BMR, basal metabolic rate; ALT, alanine aminotransferase; AST, aspartate aminotransferase; ALP, alkaline phosphatase; BUN, blood urea nitrogen; TC, total cholesterol; TG, triglycerides; HbA1c, haemoglobin A1c; SD0 baseline in the sleep deprivation group, NSD0 baseline in nonsleep deprivation group, *P < 0.05, **P < 0.01

At the end of 12 weeks after CRD intervention (Fig. 4), in the NSD group, there was a significant positive correlation between Akkermansia and FBG. Bifidobacteriaceae was positively related to FBG, HbA1C and TG. Clostridiales was negatively related to BUN. There was a negative correlation between Mollicutes and BMR. However, in the SD group, Burkholderiales was positively related to FBG. Desulfovibrionaceae was positively related to AU. Actinobacteria and Actinomycetaceae were negatively related to VFA.

### Alpha diversity of Mollicutes in the two groups before and after the intervention

**Fig. 5:**
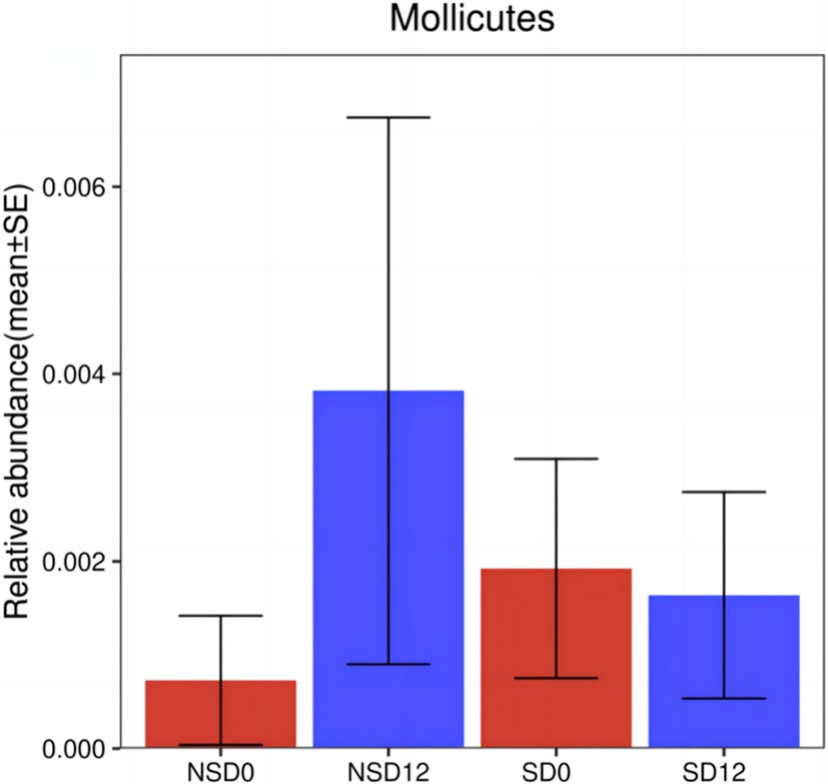
Alpha diversity of Mollicutes in the two groups before and after the intervention. P_SD0–SD12_, the change in Mollicutes in the SD group after 12 weeks of intervention. PNSD0–NSD12, the change in Mollicutes in the NSD group after the 12-week intervention. SD0: baseline in the SD group, NSD0: baseline in the NSD group, SD12: the end of 12 weeks after CRD intervention in the SD group, NSD12: the end of 12 weeks after CRD intervention in the NSD group. *P < 0.05

A stable negative correlation between Mollicutes bacteria and BMR was noted in the NSD group before and after the CRD intervention (Figs. 3, 4). To better analyse the relations between Mollicutes and BMR, Mollicutes Alpha diversity was analysed separately in Fig. 5 There were no significant differences in the relative abundance of Mollicutes before and after the intervention in either the SD group or the NSD group (*P*SD0–SD12 = 0.685, *P*NSD0–NSD12 = 0.524). However, the change in the relative abundance of Mollicutes in the NSD group after the intervention was significantly greater than that in the SD group.

## Discussion

Obesity is a risk factor for many diseases, including type-2 diabetes mellitus (T2DM), nonalcoholic fatty liver disease and cardiovascular disease [14]. Therefore, obesity is a global public health problem that should be actively managed. This study showed that after 12 weeks of a CRD and exercise intervention, some clinical parameters, including WC, BW, SBP, DBP, BFC, BMR, HOMA-IR, TG and ALT, significantly decreased. This suggests that a CRD intervention can effectively reduce BW and BFC, improve fat metabolism and relieve insulin resistance even in the presence of SD. Although this has been reported previously [15, 16], findings in previous studies were from patients who were diagnosed with simple obesity, and the CRD program was designed by a professional nutritionist and a fitness professional before the study. Therefore, the results may be helpful to improve the management of obesity.

Studies have indicated that SD may promote the occurrence and development of obesity in children and adults [17, 18]; however, the relationship between SD and weight loss is poorly understood. This study showed that a CRD intervention had a good effect on weight loss in all subjects and improved WC, BW, BMR, SBP and DBP. When subjects divided into the SD and NSD groups, BFC and TC were significantly reduced in the NSD group after the intervention, while both remained unchanged in the SD group. In addition, the changes in VFA, BW, BFC, BMR and TC in the NSD group were more obvious than those in the SD group after the CRD intervention. This further indicates that SD compromises the protective effect of a CRD intervention on weight loss and lipid metabolism. Moreover, the prevalence of fatty liver disease in the NSD group and SD group decreased after weight loss following the 12-week intervention, and 87.5% of subjects in the NSD group had no fatty liver disease, which was significantly higher than that in the SD group (37.5%). Therefore, SD may also Compromise the improvement of fatty liver disease during CRD intervention. However, the specific mechanism is still unclear. There are several possible explanations: (1) SD may lead to uncontrollable behaviours and poor diet compliance in the process of weight loss [17, 19]; (2) sleep deprivation may decrease leptin and increase ghrelin as well as hunger and appetite, which may cause a decrease in the basal metabolic rate and an increase in intake, ultimately leading to obesity [20]; and (3) SD may damage the composition of the gut microbiota [21], thereby increasing intestinal permeability, compromising the metabolism of nutrients such as fat and sugar and finally resulting in low energy expenditure and ultimately obesity [22]. Among these mechanisms, the most remarkable one is that SD damages the diversity of gut microbiota and thus compromises the protective effects of a CRD intervention.

To further investigate the potential mechanism, the gut microbiota was detected before and after the CRD intervention in all subjects. Studies have reported that diet can quickly and effectively change the composition of the gut microbiota [23, 27]. However, no significant differences were found in the alpha diversity analysis of intergroup differences in gut microbiota (Fig. 2A). However, the abundance of the curve of gut microbiota at baseline and the end of 12 weeks after intervention changed to a certain extent. The results showed that after 12 weeks of intervention, the abundance of gut microbiota in the NSD group was the highest, while that in the SD group showed almost no change. The reason why the gut microbiota abundance of the NSD group after CRD intervention was higher than that of the SD group might be that SD may gradually compromise the rapid regulatory effect of diet on the gut microbiota [24].

In addition, the relationships between gut microbiota and clinical factors were evaluated in all subjects. There were close correlations between the species abundance of the gut microbiota and clinical factors, and the diversity of the gut microbiota in the SD group was inhibited at baseline and had no significant relationship with clinical factors. Most of these correlations were unstable and nonsignificant, but a stable negative correlation between Mollicutes bacteria and the BMR was noted in the NSD group before and after CRD intervention. Compared with the SD group, the relative abundance of Mollicutes increased significantly in the NSD group. However, there was no similar stable correlation between the BMR and the abundance of Mollicutes in the SD group. Therefore, it is speculated that SD disturbs the dynamic balance of gut microbiota, which leads to the stable correlation between the BMR and abundance of Mollicutes being broken. A study has shown that the recovery of a low BMR is a sign of better weight loss [25]. Therefore, in obese patients without SD, increasing the abundance of Mollicutes may be beneficial to reduce the BMR and improve the weight loss effect of CRD. However, the specific mechanism should be elucidated in future studies.

This study also showed that a CRD intervention reduced SBP and DBP in obese subjects with or without SD, but the change in DBP in the NSD group was more obvious than that in the SD group. A study suggested that SD compromises the antihypertensive effect of a CRD intervention because of the blood pressure-increasing effect of SD [26]. Interestingly, the change in SBP in the SD group was more obvious than that in the NSD group. This might be ascribed to the higher SBP at baseline in the SD group; thus, the BP was sensitive to a dietary intervention, but this should be further confirmed in more studies.

### Strength and limits

This study is the first to investigate the relationships among SD, CRD interventions and gut microbiota. It suggests that targeting the gut microbiota in obese patients could be the key to an effective treatment on obesity. Unfortunately, there were limitations in our study. (1) This was a single-centre, prospective clinical study with a small sample size. (2) The CRD intervention was administered for only 12 weeks, which was relatively short. Therefore, more studies with large sample sizes are needed to confirm our findings and investigate the effects of a long-term CRD intervention in obese subjects, which may provide a new strategy for the clinical management of obesity.

### What is already known on this subject

Previous research has shown that the CRD intervention yields notable reductions in fat content and weight loss, and during this intervention, the modulation of gut microbiota composition has the potential to improve obesity. One of the key determinants influencing the onset and progression of obesity is the impact of sleep deprivation (SD), which possesses a notably greater significance. This factor has the potential to disrupt the efficacy of weight reduction efforts through its ability to modify the composition of the gut microbiota.

### What does this study add?

This study represents the initial investigation linking CRD, SD, and gut microbiota. It elucidates the potential detrimental impact of SD on weight loss in obese patients undergoing CRD treatment while highlighting the significant role of Mollicutes bacteria in the CRD intervention for obese patients with SD.

## Conclusions

In conclusion, the results indicate that a CRD intervention may effectively reduce BW, WC, BMR, SBP and DBP of obese subjects and improve lipid metabolism in subjects with normal sleep rhythms. SD may impair the weight loss effects of a CRD intervention and inhibit the diversity of gut microbiota in nondiabetic obese subjects. Our research suggests that Mollicutes bacteria may be an important factor affecting the weight loss effect of a dietary intervention in obese patients with SD. Further research on Mollicutes may provide an important basis for the future treatment of obesity by targeting the gut microbiota.

### Supplementary Information

Below is the link to the electronic supplementary material.

Supplementary file1 (DOCX 69 KB)

## Data Availability

The data sets during and/or analysed during the current study are available from the corresponding author on reasonable request.

## Declarations

### Statement

A preprint of this article [Surong Wen et al. 2021] has been presented on the Research “research Square” website [27].
