# Supplementary material for: Effects of a calorie-restricted dietary intervention on weight loss and gut microbiota diversity in obese patients with sleep deprivation
Source: Eat Weight Disord. 2023 Oct 4;28(1):80. doi: 10.1007/s40519-023-01609-5 (PMC10550869; doi:10.1007/s40519-023-01609-5)
Supplement: Supplementary file 1 — Supplementary file1 (DOCX 69 KB) [file 40519_2023_1609_MOESM1_ESM.docx]

| Examples of doppler ultrasound image of fatty liver | | |
| --- | --- | --- |
| 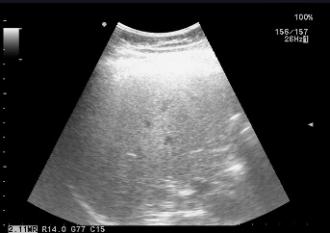 | 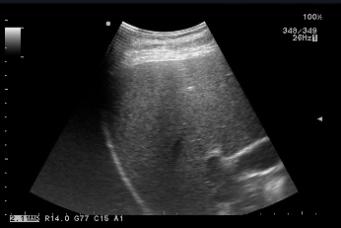 | 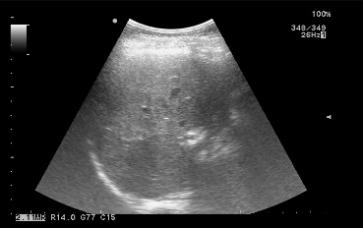 |
| Examples of doppler ultrasound image of normal liver | | |
| 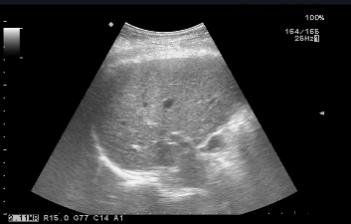 | 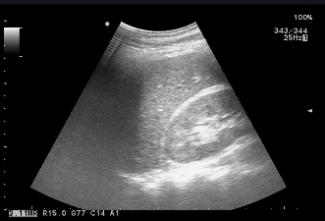 | 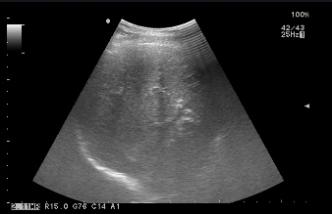 |
